# Molecular Detection and Phylogenetic Analysis of Endosymbiont *Wolbachia pipientis* (Rickettsiales: Anaplasmataceae) Isolated from *Dirofilaria immitis* in Northwest of Iran

**Published:** 2019-03-30

**Authors:** Majid Khanmohammadi, Reza Falak, Ahmad Reza Meamar, Mehdi Arshadi, Lame Akhlaghi, Elham Razmjou

**Affiliations:** 1 Department of Parasitology and Mycology, School of Medicine, Iran University of Medical Sciences, Tehran, Iran; 2 Department of Laboratory Science, Marand Branch, Islamic Azad University, Marand, Iran; 3 Immunology Research Center, Iran University of Medical Sciences, Tehran, Iran; 4 Department of Immunology, School of Medicine, Iran University of Medical Sciences, Tehran, Iran; 5 Al-Zahra Hospital, Tabriz University of Medical Sciences, Tabriz, Iran

**Keywords:** *Wolbachia pipientis*, *Dirofilaria immitis*, Cytochrome oxidase I (*COI*), *Wolbachia* surface protein (*WSP*), Phylogenetic analysis

## Abstract

**Background:**

The purpose of this study was molecular detection and phylogenetic analysis of *Wolbachia* species of *Dirofilaria immitis*.

**Methods:**

Adult filarial nematodes were collected from the cardiovascular and pulmonary arterial systems of naturally infected dogs, which caught in different geographical areas of Meshkin Shahr in Ardabil Province, Iran, during 2017. *Dirofilaria immitis* genomic DNA were extracted. Phylogenetic analysis for proofing of *D. immitis* was carried out using cytochrome oxidase I (*COI*) gene. Afterward, the purified DNA was used to determine the molecular pattern of the *Wolbachia* surface protein (*WSP*) gene sequence by PCR.

**Results:**

Phylogeny and homology studies showed high consistency of the *COI* gene with the previously-registered sequences for *D. immitis*. Comparison of DNA sequences revealed no nucleotide variation between them. PCR showed that all of the collected parasites were infected with *W. pipientis*. The sequence of the *WSP* gene in *Wolbachia* species from *D. immitis* was significantly different from other species of *Dirofilaria* as well as other filarial species. The maximum homology was observed with the *Wolbachia* isolated from *D. immitis*. The greatest distance between *WSP* nucleotides of *Wolbachia* species found between *D. immitis* and those isolated from *Onchocerca lupi*.

**Conclusion:**

PCR could be a simple but suitable method for detection of *Wolbachia* species. There is a pattern of host specificity between *Wolbachia* and *Dirofilaria* that can be related to ancestral evolutions. The results of this phylogenetic analysis and molecular characterization may help us for better identification of *Wolbachia* species and understanding of their coevolution.

## Introduction

*Wolbachia* is an intracellular α-proteobacteria, endosymbiont in insects and filarial nematodes. This genus of bacteria belongs to the phylum of Proteobacteria and order of Rick-ettsiales. *Wolbachia pipientis* was the first bacteria discovered in this genus, which infects *Culex pipiens* mosquito ovaries (1, 2) and plays a key role in the mosquito’s biology, ecology, immunity, evolution, parthenogenesis, microbial manipulation and reproduction (3, 4). This intracellular bacterium provides mechanisms to destroy male embryos and increase feminization of the filarial host, thus it is characterized by reproductive parasitism (3, 4). During feminization, these bacteria affect various biological characteristics including female worm fertilization, molting, development, survival, and killing of the male hosts. Most likely, a cytoplasmic incompatibility occurs and results in this phenomenon, however, up to now no definitive proof was found (4, 5).

*Wolbachia* has a role in filarial nematode development, too. It mainly affects host’s reproductive system, increases the transfer of nematode larva from the insect to the mammalian host, affects embryogenesis, promotes progression of the L_1_ form to the L_3_ form, reduces conversion time, and speeds up transition of the third-stage larva to adult forms (6). *Wolbachia* infection of the filarial nematodes may also cause mild inflammatory responses in mammalian hosts, too. This intracellular bacterium is mostly located in the lateral hypodermal cords of both male and female nematodes. In this way, the worms may transmit their eggs to the cytoplasm of the female reproductive structures (7). Unfortunately, data regarding the symbiotic relationship between *W. pipientis* and *D. immitis* is limited; thus, the role of these bacteria in the pathogenesis of *Dirofilaria*, as the causative agent of canine heartworm disease, is not fully understood. However, some data support the role of this bacterium in the pathophysiology and survival of distinct pathogenic filarial worms including *O. volvulus*, *W. bancrofti*, *B. malayi*, *D. repens*, and *D. immitis* (8–10). Therefore, capability of this endosymbiont bacterium in manipulation of the reproductive system of the host could be beneficial for biological control and management of pests or for in molecular identification of the parasites.

*Wolbachia* spp. could be a vaccine candidate against vector-borne diseases including dengue and filariasis, too (3, 4). Antimicrobial therapy against *Wolbachia* spp. may be useful in treatment of heartworm disease in dogs through decreasing the number of endogenous *Wolbachia* and consequently the microfilarial load, inhibition of the larval stage and also worm infertility. Typically, application of antimicrobial substances against *Wolbachia* may be useful as a complementary anti-filarial therapy strategy in canine and feline dirofilariasis, but further studies are needed to substitute these novel methods for anti-parasite drugs (11). In this regard, doxycycline is an effective treatment against *Wolbachia*, and in combination with ivermectin has been shown to have adulticidal efficiency for heartworm treatments in dogs (12–15). *Wolbachia* metabolic products may also exert pathological changes in many organs of the host such as lungs and kidneys (16).

The *Wolbachia* surface protein (*WSP*) is a crucial component involved in the immuno-pathogenesis of filarial diseases and helps in parasite evasion from potentially harmful immune responses of the host (16). *Dirofilaria immitis* is a selective reservoir for *W. pipientis*, therefore, this bacterium provides attenuation strategy for *Dirofilaria* to evade host immune responses (17, 18). *Wolbachia* is effective in generating immune responses during heart-worm infections; thus, *Wolbachia* likely influences the inflammatory and immunoregulatory functions of the host (19). Therefore, its control may offer therapeutic and diagnostic possibilities. Elimination of *Wolbachia* by antimicrobial agents can exert a preventive effect on embryogenesis of *D. immitis* and could have potential application in sterilization of the female worms and consequently control and treatment of dirofilariasis (16). Interfering of this obligate relationship has been used in novel therapeutic strategies; for example, a combination of antimicrobial therapy using doxycycline and ivermectin, as a macrocyclic lactone can destroy nematodes (8, 11, 14). Overall, due to the zoonotic potential and the increasing number of *D. immitis* and *D. repens* infections in non-endemic areas, xenomonitoring of this bacterium may be useful (20).

In Iran, few studies have focused on detection of *Wolbachia* species or their invertebrate hosts. Herein, we performed molecular detection and phylogenetic analysis of *Wolbachia* in *D. immitis*, using the *WSP* gene. The main reasons for selecting *WSP* gene in this study was the high availability of the corresponding protein in soluble and membranous forms in *Wolbachia* species, the feasibility of the amplifying its coding sequence by PCR, considerable dissimilarity of its expression in different filarial genus, and its applicability as an indicator of parasite evolution and bacterial phylogenic proximity (21).

## Materials and Methods

Forty-three stray domestic dogs (*Canis familiaris*) suspicious to dirofilariasis were collected during 2017 from different geographical areas of Meshkinshahr, Ardebil Province, Iran. Meshkinshahr (38°23′56″N 47°40′55″E) is a main endemic area for canine dirofilariasis in Iran. All dogs were tested for dirofilariasis by direct microscopy, ELISA, and rapid dip-stick methods. Direct wet smears of whole blood were taken with EDTA and used for detection of microfilaria by light microscopy. Sera was analyzed for adult *D. immitis* circulating antigen using a commercial ELISA kit (DiroCHEK®, USA) as well as dipstick method (SNAP® 4Dx® Test, CHW II kit IDEXX Laboratory, USA) according to the manufacturers’ instructions.

All procedures were carried out in admission with the rules and regulations of the respective national animal Ethics Committee of Iran University of Medical Sciences (IR. IUMS.REC1395.9221577203-2016.05.09).

Six hyper-infected dogs euthanized and necropsied. Worms were collected from the pulmonary arteries and right ventricles. Then, their DNA was extracted and used for molecular analyses.

### DNA extraction

Adult worms were homogenized by a rotor-stator system (IKA, UK) and total genomic DNA was extracted using DNA extraction kit (QIAGEN GmbH, Germany) from approximately 25mg of each *Dirofilaria* sample, according to the manufacturer’s instruction. Purity of the extracted DNA was determined and DNA samples stored at −20 °C. For amplification of cytochrome oxidase I gene (*COI*) which is a species-specific mitochondrial gene of *D. immitis*, a set of specific primers were designed. In details, 5′-TGA TTG GTG GTT TTG GTA A-3′ and 5′-ATA AGT ACG AGT ATC AATATC-3′ were used as forward and reverse primers, respectively. In addition, we used *Wolbachia*-specific primers to amplify nucleotides 81–691 of *WSP*. The forward and reverse primers were 5′ TGGTCCAATAAGTGATGAAGAAAC-3′ and 5′ AAAAATTAAACGCTACTCCA-3′, respectively. Reactions were performed in 25μl volumes using 2x PCR Master Mix (RED Amplicon, Denmark), 1μl of DNA template, and 1μl of each primer. The thermal cycler program included one cycle of 94 °C for 5min followed by 35 cycles of 94 °C for 1min (denaturation), 55 °C for 1min (annealing), 72 °C for 60sec (extension), and a final extension at 72 °C for 10min (22). PCR products were electrophoresed on 1.5% agarose gel along with a commercial DNA marker (SMOBiO DM3100) using an imaging system (SYNGENE, UK). Finally, PCR products were purified using the PCR purification Kit (QIAGEN GmbH, Germany) and directly subject for sequencing.

### Molecular and phylogenetic analysis

Phylogenetic analysis of *D. immitis* was carried out using *COI* gene sequences, obtained during the study (Accession code, MF288560.1 Mesh-Iran 3) along with previously determined relevant sequences in the GenBank. Then, all obtained nucleotide sequences were analyzed and compared with the available complete *WSP* sequence in GenBank (MG010709.1) using BioEdit software, ver. 7.0.5 (California, USA). All of the evolutionary aspects and other molecular data were inferred using the maximum likelihood method, based on the Tamura-Nei model (23). The phylogenetic tree was drawn to scale, with branch lengths measured as the number of *Wolbachia* substitutions per site and with the highest log likelihood. Multiple alignments and sequences linking functionality of phylogenetic studies were analyzed with MEGA7 software (24). The accuracy of the phylogenetic tree was measured by 1000 bootstrap replication. Nucleotide changes were compared to the reference sequence with Sequencher software (Sequencher® version 5.4.6 USA).

## Results

We examined blood samples from 43 dogs with obvious symptoms of dirofilariasis and found that 27 dogs were Dirofilaria seropositive (62.8% CI: 47.9 to 75.6). DNA was purified from the collected worms and used as PCR template. *Wolbachia* DNA was detected in all 67 filarial worms including 41 females and 26 male parasites.

**Fig. 1.**
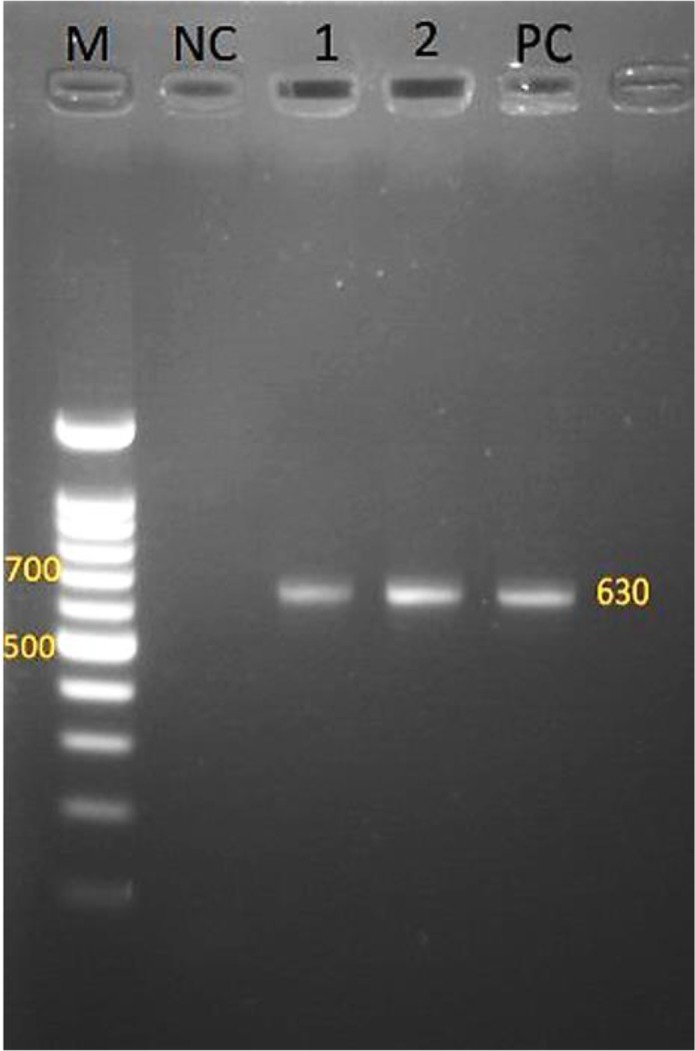
Electrophoresis of PCR product of *WSP* specific amplification confirming the presence of endosymbiotic *Wolbachia* present in *Dirofilaria immitis* DNA extract. M: 100bp DNA marker, NC: Negative control, Lines 1–2: PCR products, PC: Positive control

**Fig. 2.**
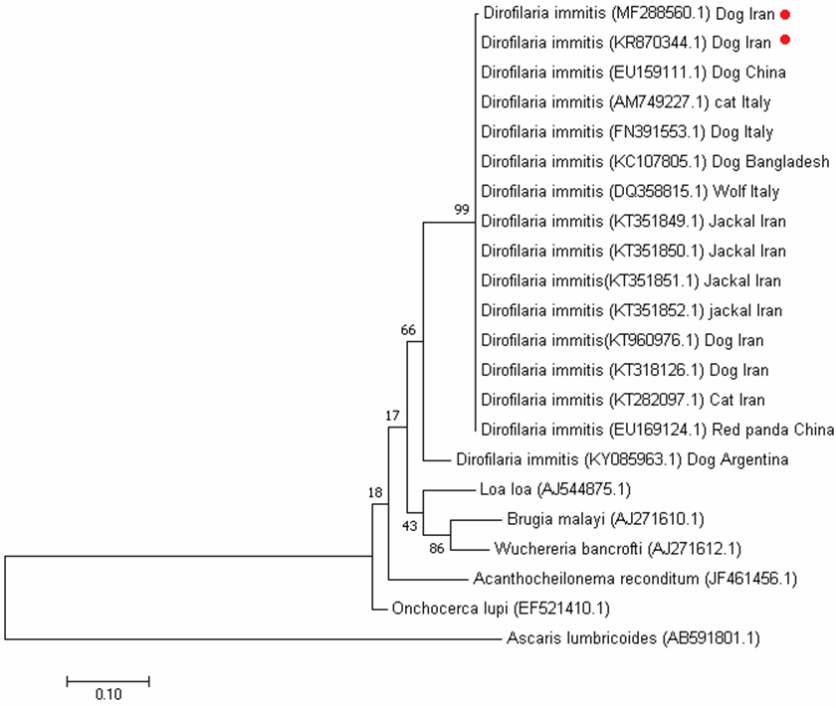
Phylogenetic tree of *Dirofilaria immitis* cytochrome c oxidase subunit I (*COI*) gene using maximum likelihood analysis among sequences based on the Tamura-Nei model with 1000 bootstrap repetition. The scale bar indicates the genetic distance in single nucleotide substitutions. GenBank registered accession numbers are specified within parentheses. *Ascaris lumbricoides* (Accession no. AB591801.1) was as outgroup

Electrophoresis of PCR product of the amplified *WSP* gene confirmed presence of a 630bp *Wolbachia* specific sequence in all of the DNA extracts (Fig. 1). The BLASTn analysis of the *COI* gene indicated maximum homology of the isolated parasites’ DNA with previously isolated sequences of *D. immitis*. Phylogenetic analysis showed highest homology with *D. immitis* isolated from dogs in Kerman City (KR 870344.1). We also found good homology with *D. immitis* isolated from several other geographical areas such as those dogs from Iran (KT960976.1, KT318126. 1), China (EU159111. 1), Italy (FN391553. 1) and Bangladesh (KC107805.1), and also other animals such as jackal (*Canis aureus*) in North Khorasan of Iran (KT351850.1, KT351851.1, KT351852. 1), cat from Iran (KT282097. 1) and Italy (AM749227. 1), wolf from Italy (DQ358815. 1) and red panda from China (EU169124.1) (Fig. 2). Sequence analysis in all samples demonstrated a DNA band which belonged to *WSP* gene of *W. pipientis*. Sequence similarities and homologies between the amplified one and the previously registered sequences in the GenBank was determined following a basic local alignment sequence analysis, and calculating the statistical significance through the National Center for Biotechnology Information (NCBI) (https://blast.ncbi.nlm.nih.gov/Blast.cgi). Multiple sequences were aligned with the online clustalW2 tool (http://www.ebi.ac.uk/Tools/msa/clustalw2). All obtained sequences were blasted after alignment and belonged to *W. pipientis*. Partial *D. immitis WSP* nucleotide sequences were submitted with accession code of MG010709.1 in GenBank.

**Fig. 3.**
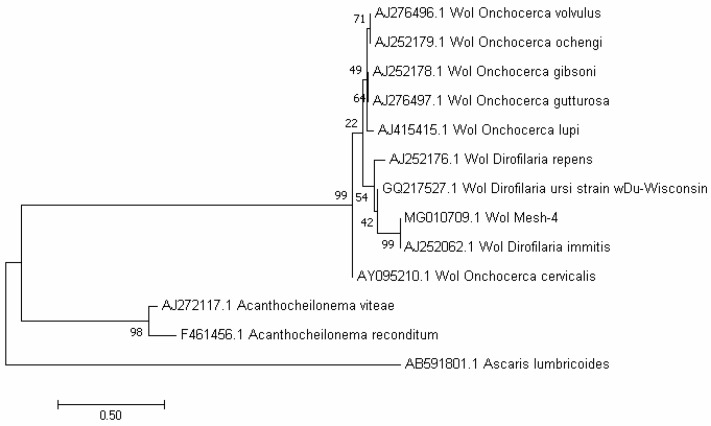
Phylogenetic tree of *Wolbachia WSP* gene sequences from Meshkinshahr of North West Iran and other previous registered sequences from different areas using maximum likelihood method based on the Tamura-Nei model with 1000 bootstrap repetition

The phylogenetic analysis of *WSP* sequences of *Wolbachia* showed maximum homology (99%) with the complete *W. pipientis* sequence isolated from *D. immitis* in Italy (AJ252062.1) and several other filarial organisms from different areas of the world including *D. repens* (AJ252176.1), *O. volvulus* (HG810405.1), *O. ochengi* (HE660029.1), *O. cervicalis* (AY 095210.1), *O. gibsoni* (AJ252178.1), and *O. gutturosa* (AJ276497.1) (Fig. 3).

*Wolbachia pipientis* is genetically related to the filarial nematodes phylum. Phylogenetic studies using *WSP* revealed the greatest similarity and proximity of the obtained sequence with other filarial nematodes. The least similarity of the *Wolbachia* sequence isolated from filarial nematodes was observed between *Onchocerca* and *Dirofilaria* taxa (Fig. 3).

**Fig. 4.**
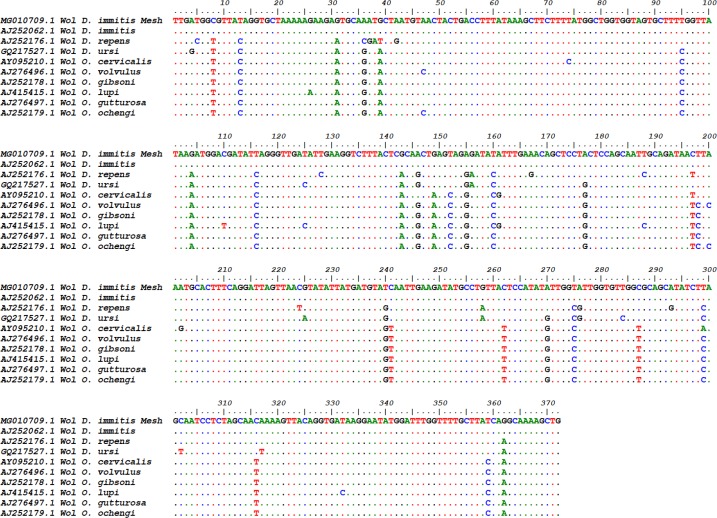
Multiple alignments of the partial *WSP* gene and the sequences of other filarial nematodes

Nucleotide changes were compared and no differences were found between the amplified sequences and the previously reported ones. The highest sequence similarity with *W. pipientis* was seen in *O. gibsoni* and *O. gutturosa*, and the lowest was in *O. lupi* due to difference in 33 nucleotides. The nucleotide difference between *D. immitis* and *D. repens* was found in 27 nucleotides. The difference between *D. repens* and the other filarial nematodes were lowest of those studied ones (Fig. 4). All studied species showed the greatest nucleotide differences between positions 143 and 161 of the known nucleotide location. This study showed sequence variation in *WSP* gene of *Wolbachia* from various filarial nematode population.

## Discussion

This was the first report on molecular characterization of *W. pipientis* infection of *D. immitis* isolated from northwest of Iran, where, dirofilariasis is endemic. The prevalence of dirofilariasis in the untreated dogs was previously estimated to be in the range of 1.4–51.4% (25, 26). Results of phylogenetic analysis of *COI* gene in the isolated filarial nematodes confirmed that the *Dirofilaria* species in Kerman and Meshkinshahr are molecularly identical. Pairwise homology analysis revealed high sequence homology of the isolated worms with the available sequences at NCBI GenBank. The determined *Wolbachia* infection rate was very similar to previous studies. The findings also confirmed the general believe about *Wolbachia* incidence and emphasized the absence of ancestral infection in the nematode phylum (27).

This widespread bacterium may be detected in all classes of arthropods and approximately infects 20–70% of them. Therefore, due to high incidence of the endosymbiosis infection of the arthropods with *Wolbachia*, recent studies were mainly focused on arthropods (28). In brief, it is commonly found in butterflies, fruit flies, sand flies, mosquitoes, fleas, ticks, bugs, wasps, silkworms (4, 5, 19, 21, 29–34). Previously, *Wolbachia* DNA was detected in 30.6% of the blood sample of filaria-positive dogs in the Mediterranean area (35). Co-infection of six dogs with *D. immitis* and *D. repens* was reported in Turkey by PCR-amplification of the *WSP* gene (36). *Wolbachia* DNA was also found in blood samples from *Dirofilaria*-infected dogs, in other regions (37). In a Portuguese study, dogs whose dirofilariasis was confirmed by parasitological and serological methods were further examined by PCR method. Moreover, *Wolbachia* DNA was detected in blood sample of 52.6% of those with occult infection. In occult infections, no obvious microfilaria could be found in microscopic examination of blood. This molecular evaluation is useful because the presence of *Wolbachia* in association with microfilaria is critical for control of canine dirofilariasis (38). Anti-*WSP* IgG titer in urine samples from 19 *Dirofilaria-*infected dogs was greater than the established cut-off values, and *WSP* gene was detected in kidney glomerular capillaries in those that were seropositive, too (39).

*Wolbachia* has also previously been isolated from *W. bancrofti*, *Brugia malayi*, *O. volvulus*, and *Setaria tundra* (29, 40–42). Recently, the study of the isolated *O. volvulus* from 4 African countries indicated that extensive intra species heterogeneity exists in the *Wolbachia* content of male and female adults of *O. volvulus*. *Wolbachia* variation was previously reported in some studies and was in line with our results and pointed out to the host specificity of this bacterium (43). Diversity of the pattern of the mitochondrial genetic content was found in *D. immitis* and was much lower than the related species (44).

Phylogenetic studies confirmed homology of the amplified sequence with a previously registered *Wolbachia* sequence in GenBank. We found the most consistency with *Wolbachia* species isolated from *D. immitis* in Italy (45). Filarial nematodes have gained this bacterium endosymbiosis from arthropods, and the current data indicates that this bacteria has spread globally in filarial nematodes (46). We hypothesized that *Wolbachia* has a genetic relationship with the phylum of filarial nematodes and this association was most likely generated over times.

Sequence comparisons between *Dirofilaria* species and other filarial nematodes revealed difference of several nucleotides in *WSP* gene. Compression of the sequences from our study with previously-reported *Wolbachia* sequences indicated that the amplified sequence is likely *Dirofilaria*-specific. Interestingly, BLASTn studies showed a specific *WSP* pattern in *Dirofilaria*. This could be due to genetic characteristics of the endosymbiont bacteria, as well as the physiological properties and adaptation of *Dirofilaria*, and the regulatory mechanisms of the immune system of the final host.

Many questions still remain unanswered in this field. Regarding to the reported distribution of *Wolbachia* in filarial nematodes, it is not clear whether the absence of *W. pipientis* in *Wolbachia*-negative filarial nematodes represents an ancestral characteristic of this bacteria or is due to losing the inserted sequence over time due to low transpositional activity in some filarial species (41). The phylogenies of various filarial nematodes are still not identified completely; thus, it is difficult to express the presence or absence of *W. pipientis* on a tree indicating filarial genetic evolution (42). The presence of such extensive proprietary pattern between adult worms of both sexes in filarial nematodes provides questions regarding the specific symbiosis between the *Wolbachia* and filarial worms, and the mechanisms by which *Wolbachia* is involved. Finally, our results suggest that *W. pipientis* isolated from Iranian *Dirofilaria* has distinct genomic features, which likely are the result of long periods of independent evolution. The results of this study open the way for further studies on the strain identification and genetic diversity, which may increase our understanding about the host-parasite relationships. Overall, the mapping of *Wolbachia* on the phylogenetic trees generated by Mega software indicates that this bacterium may have evolved along with ancestors of filarial nematodes.

## Conclusion

Main differences between the population of adult filarial worms were at the level of *Wolbachia* species. There is a pattern of host specificity between *Wolbachia* and *Dirofilaria*. This subject can be due to ancestral evolutions long times ago. Molecular characterization can be applied as a new trend for understanding the evolution and identification of *Wolbachia*. This method offers a new technique for diagnosis and may provide strategies for development of novel and effective therapeutic procedures. The results of this study can help us to understand the interactions of *Wolbachia* and *Dirofilaria* with their mammalian hosts. We suggest further investigation of the endosymbiotic *W. pipientis* polymorphisms in nematodes. Findings also highlighted correlations between *Wolbachia* and *D. immitis* throughout their life cycle and could be a valuable resource that may be applied for providing novel intervention strategies, not only for the treatment and prophylaxis of dirofilariasis but also in other closely related human filarial disease as well.
